# Synergistic Effect of Lupenone and Caryophyllene Oxide against *Trypanosoma cruzi*

**DOI:** 10.1155/2013/435398

**Published:** 2013-05-20

**Authors:** Glendy Polanco-Hernández, Fabiola Escalante-Erosa, Karlina García-Sosa, María E. Rosado, Eugenia Guzmán-Marín, Karla Y. Acosta-Viana, Alberto Giménez-Turba, Efraín Salamanca, Luis M. Peña-Rodríguez

**Affiliations:** ^1^Unidad de Biotecnología, Centro de Investigación Científica de Yucatán, Calle 43 No. 130, Colonia Chuburná de Hidalgo, 97200 Mérida, YUC, Mexico; ^2^Centro de Investigaciones Regionales “Dr. Hideyo Noguchi”, 97200 Mérida, YUC, Mexico; ^3^Instituto de Investigaciones Fármaco Bioquímicas, Universidad Mayor de San Andrés, La Paz, Bolivia

## Abstract

The *in vitro* trypanocidal activity of a 1 : 4 mixture of lupenone and caryophyllene oxide confirmed a synergistic effect of the terpenoids against epimastigotes forms of *T. cruzi* (IC_50_ = 10.4 **μ**g/mL, FIC = 0.46). In addition, testing of the terpenoid mixture for its capacity to reduce the number of amastigote nests in cardiac tissue and skeletal muscle of infected mice showed a reduction of more than 80% at a dose level of 20.8 mg·kg^−1^·day^−1^.

## 1. Introduction

Chagas disease is a chronic parasitosis caused by the flagellate protozoan *Trypanosoma cruzi, *which is transmitted by an insect vector of the *Reduviidae* family causing cardiac injury leading to death [1]. The disease represents an important public health problem in Latin America, with an estimated 10 million people infected and 25 million people under risk of infection [2]. At present, there is no satisfactory chemotherapy for the disease also known as American trypanosomiasis; the drugs currently used, which include nifurtimox, benznidazole, and allopurinol, are toxic, have severe side effects, and are effective mainly in the acute phase, while their activity in the chronic phase of the disease is low and controversial [3–5]. Because of this, the WHO has emphasized the need to develop new and better trypanocidal drugs with none or limited side effects [6].

A strategy for the development of new and more efficient pharmaceuticals is to evaluate the synergism between two or more products as part of a treatment of combined therapy. Often, the therapeutic activity of a combination of drugs is greater than the activity of each product when administrated separately; additionally, synergism can improve the efficiency of the treatment, broaden its spectrum of action, limit the development of resistant strains, and reduce its duration and toxicity [7, 8]. These arguments, supported by the recent recommendation by the WHO that oral artemisinin-based monotherapies are withdrawn from the market and replaced with artemisinin-based combination therapies for the treatment of malaria [9], emphasize the importance of considering combined therapies as an alternative for the treatment of protozoan diseases.

We have recently carried out a study of native plants of the Yucatán Peninsula and reported the presence of trypanocidal activity in the leaf extract of *Serjania yucatanensis* [10]. The bioassay-guided purification of the bioactive crude extract resulted in the identification of a 1 : 1 mixture of terpenoids, lupenone (a triterpene) and caryophyllene oxide (an oxygenated sesquiterpene), as that responsible for the originally detected trypanocidal activity; the mixture also proved to inhibit the egress of trypomastigotes from infected Vero cells without being cytotoxic [11]. We wish to report herein on the synergism of a 1 : 4 mixture of lupenone and caryophyllene oxide when tested *in vitro* for trypanocidal activity and *in vivo* when tested against the amastigote form of the parasite during the chronic phase of the infection.

## 2. Materials and Methods

### 2.1. Lupenone and Caryophyllene Oxide

Commercial caryophyllene oxide (Sigma-Aldrich) and lupenone obtained from the oxidation of commercial lupeol (Sigma-Aldrich) were used in all tests. Mixtures were prepared by combining the two terpenoids in different proportions (1 : 0, 1 : 4, 2 : 3, 1 : 1, 3 : 2, 4 : 1, and 0 : 1; w/w).

### 2.2. Parasites and Their Growth Conditions

Epimastigotes forms of the Tulahuen strain and blood trypomastigote forms of the H4 strain (isolated from a patient with Chagas disease in Yucatán, Mexico) of *T. cruzi* were used in this study [12]. Epimastigotes were obtained from liver infusion tryptose medium supplemented with 10% fetal bovine serum [13] and blood trypomastigotes were obtained by successive infections of BALB/c mice.

### 2.3. Evaluation of the **In Vitro ** Trypanocidal Activity

The trypanocidal activity was assayed on epimastigotes of *T. cruzi *(Tulahuen strain). Experiments were carried out using 96-well microplates containing 1 × 10^5^ epimastigotes/mL. The different proportions of mixture of lupenone and caryophyllene oxide (1 : 0, 1 : 4, 2 : 3, 1 : 1, 3 : 2, 4 : 1, and 0 : 1) as well as the crude extract of *S. yucatanensis* and the low-polarity (hexane) fraction obtained from the crude extract [10, 11] were dissolved in dimethylformamide (DMF; final solvent concentration not greater than 1%) and were evaluated at 100, 50, 25, and 12.5 *μ*g/mL. For each experiment there were controls of parasites growing in the presence and absence of DMF. The different mixtures and their corresponding concentrations were added to the wells, and the plates were incubated at 28°C for 72 h. All assays were performed in duplicate. The activity was evaluated using the XTT colorimetric method, which is based on the reduction of the sodium salt of 2,3-bis(2metoxi-4-nitro-5-sulfophenyl-2-h-tetrazolium-5-carboxanilide) by mitochondrial dehydrogenases to produce formazan crystals [14]; 50 *μ*L of a solution of XTT/PMS (1 mg/mL XTT)/(0.001 mg/mL PMS) were added to each well, and the plates were incubated for an additional 4 h. The plates were read in an ELISA plate spectrophotometer at 450 nm. The activity is expressed as IC_50_ (*μ*g/mL). Amphotericin B was used as positive control because this polyene antibiotic has been used as a reference drug for the *in vitro* testing of crude extracts and purified natural products on *Trypanosoma* cultures [15, 16].

### 2.4. Evaluation of Synergism

Fractional inhibitory concentrations (FIC) were calculated as previously described [17]. We have FIC = FEa + FEb, where FEa = IC_50_  a + b /IC_50_ a and FEb =  IC_50_ a +b /IC_50_ b. Values of FIC < 1 indicate synergism, values = 1 indicate additive effect, and values >1 indicate antagonism.

### 2.5.  **In Vivo** Assay against Amastigotes of  *T. Cruzi*

Eight-week old BALB/c mice and trypomastigotes of *T. cruzi* H4 strain were used to assay for antitrypanosomal activity. Animals were maintained on a light-dark cycle and had access to food and water *ad libitum* during the entire assay. 

Thirty BALB/c mice weighing approximately 23 g were randomly divided into five groups (*n* = 6 each). The animals were infected with 100 trypomastigotes through intraperitoneal injection; inoculation conditions were selected based to the reported in previous studies in mice infected with *T. cruzi* in chronic phase [18, 19]. Mice were divided into five groups: negative control (CN): infected animals treated with PBS; positive control (CP): infected animals treated with allopurinol (8.5 *μ*g/g); hexane fraction (FHex): animals treated with the hexane fraction from the leaf crude extract of *S. yucatanensis* (41.6 mg·kg^−1^·day^−1^); dose 1 (D1): infected mice treated with a 20.8 mg·kg^−1^·day^−1^ 1 : 4 mixture of lupenone and caryophyllene oxide; dose 2 (D2): infected mice treated with a 41.6 mg·kg^−1^·day^−1^ 1 : 4 mixture of lupenone and caryophyllene oxide. All treatments were administered resuspended in phosphate buffer saline (PBS, NaCl 13.7 mM, KCl 2.7 mM, Na_2_HPO_4_ 4.3 mM y KH_2_PO_4_ 1.4 mM pH 7.4). Administration started after 45 days post infection, during the chronic phase; the mice in the experimental groups received each treatment orally (adjusted to 50 *μ*L per animal), every 24 hours, for 15 days.

### 2.6. Histopathology Study

Samples of cardiac tissue and skeletal muscle from groups of treated and control mice were collected and fixed in 10% formaldehyde for further processing. Paraffin embedded tissue sections were stained with hematoxylin-eosin and examined under a light microscope (40x). The number ofamastigotenests was quantified in 100 fields for each heart tissue and skeletal muscle sample. The experiments were carried out under the approval of the Bioethics Committee of Centro de Investigaciones Regionales “Dr. Hideyo Noguchi,” in Mérida, Yucatán, México.

### 2.7. Statistical Analyses

The statistical analyses were performed using Prism program 5.0 software. Data are presented as mean values ± S.D. Statistical analyses: one-way ANOVA and post hoc Tukey's test were used to compare different experimental groups (*P* < 0.05).

## 3. Results and Discussion

*In vitro* testing of the leaf crude extract of *S. yucatanensis*, the low-polarity (hexane) fraction from the crude extract, and the different proportions of lupenone and caryophyllene oxide (1 : 0, 1 : 4, 2 : 3, 1 : 1, 3 : 2, 4 : 1, and 0 : 1) against epimastigotes of *T. cruzi *(Tulahuen strain) showed that the crude extract of *S. yucatanensis* and the hexane fraction had a similar activity (IC_50_ = 74.5 and 61.5 *μ*g/mL, resp.) than that previously reported against *T. cruzi* (Y strain) [11]. Similarly, the trypanocidal activity observed for the 1 : 1 mixture of lupenone and caryophyllene oxide (IC_50_ = 80.3 *μ*g/mL; Table 1) is similar to that reported for the original mixture of terpenoids obtained in a approximate ratio of 1 : 1 from the leaf extract of *S. yucatanensis* (IC_50_ = 80.3 *μ*g/mL) [11]. However, the highest activity (IC_50_ = 10.4 *μ*g/mL) was observed for the 1 : 4 mixture of lupenone and caryophyllene oxide; the synergistic effect of this mixture of terpenoids against epimastigotes was confirmed by a potentiation FIC value of <1 (FIC = 0.46) (Table 1). It is interest to point out that the rest of the terpenoid mixtures proportions showed FIC values >1 indicating an antagonistic effect and suggesting that the mixture of terpenoids in a 1 : 4 proportion is necessary for the full expression of trypanocidal activity and that higher proportions of lupenone result in lower activity. The fact that lupenone does not show significant antiprotozoal activity, and that the activity of caryophyllene oxide is only moderate, is in agreement with reports in the literature describing a fraction containing lupenone as not showing trypanocidal activity against trypomastigotes of *T. cruzi* [20] and caryophyllene oxide with only a moderate inhibitory activity against cruzipain of *T. cruzi* [21]. However, lupenone has been reported to have anticarcinogenic activity in mouse melanoma [22] and to inhibit the protein tyrosine phosphatase 1B, an attractive target for the development of new drugs for type 2 diabetes and obesity [23]; the biological activities reported for caryophyllene oxide include antifungal [24], anti platelet aggregation [25], and not being cytotoxic to Vero and THP-1 cells [26].

**Figure 1:**
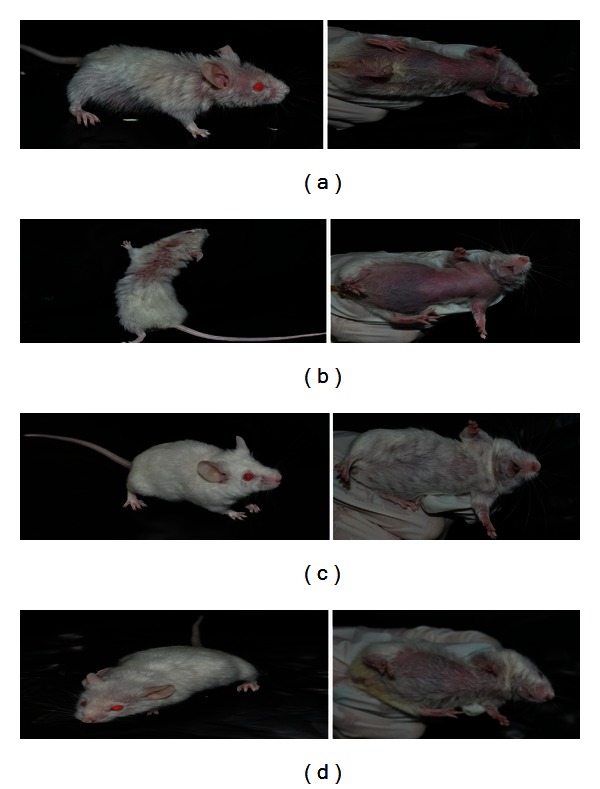
Mice belonging to the different groups: (a) negative control (CN): infected animals treated with PBS; (b) positive control (CP): infected animals treated with allopurinol (8.5 *μ*g/g); (c) dose 1 (D1): infected mice treated with a 20.8 mg·kg^−1^·day^−1^ 1 : 4 mixture of lupenone and caryophyllene oxide; (d) dose 2 (D2): infected mice treated with a 41.6 mg·kg^−1^·day^−1^ 1 : 4 mixture of lupenone and caryophyllene oxide.

**Figure 2:**
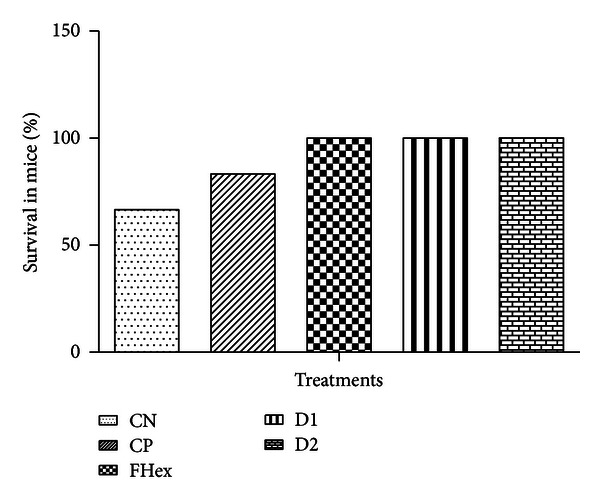
Survival rates of the five groups of *T. cruzi*-infected mice during the *in vivo* treatment: CN: negative control, infected animals treated with PBS; CP: positive control, infected animals treated with allopurinol (8.5 *μ*g/g); FHex: hexane fraction, animals treated with the hexane fraction from the leaf crude extract of *S. yucatanensis* (41.6 mg·kg^−1^·day^−1^); D1: dose 1, infected mice treated with a 20.8 mg·kg^−1^·day^−1^ 1 : 4 mixture of lupenone and caryophyllene oxide; D2: dose 2, infected mice treated with a 41.6 mg·kg^−1^·day^−1^ 1 : 4 mixture of lupenone and caryophyllene oxide.

Having the synergism of lupenone and caryophyllene oxide confirmed and taking into account that, to date, there are no reports on their combined trypanocidal activity, the 1 : 4 mixture was evaluated *in vivo* at two different doses, 20.8 mg·kg^−1^·day^−1^ and 41.6 mg·kg^−1^·day^−1^, against amastigotes, the parasite form present in the mammalian host and the one responsible for maintaining the infection. The first, most evident results observed were that mice treated with both doses of the 1 : 4 mixture of lupenone and caryophyllene oxide did not show the physical deterioration observed in mice belonging to the negative control group (i.e., infected animals only treated with PBS; Figures 1(a)–1(d)), which presented the clinical signs commonly observed in BALB/c mice infected with *T. cruzi *[27], namely, adinamia and alopecia in neck and chest. Additionally, the group treated with both doses of the 1 : 4 mixture of lupenone and caryophyllene oxide showed a better survival rate (100% of survival after 60 days of infection, Figure 2) than that observed in the positive control (83% of survival, Figure 2), which proved to be only slightly higher than that of the negative control (66% of survival; Figure 2).

**Figure 3:**
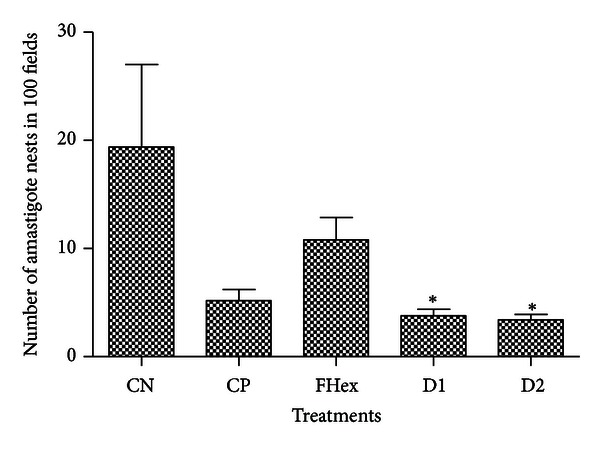
*In vivo* antitrypanosomal activity of the different treatments, determined by the number of amastigote nests observed in cardiac tissue from infected mice. CN: negative control, infected animals treated with PBS; CP: positive control, infected animals treated with allopurinol (8.5 *μ*g/g); FHex: hexane fraction, animals treated with the hexane fraction from the leaf crude extract of *S. yucatanensis* (41.6 mg·kg^−1^·day^−1^); D1: dose 1, infected mice treated with a 20.8 mg·kg^−1^·day^−1^ 1 : 4 mixture of lupenone and caryophyllene oxide; D2: dose 2, infected mice treated with a 41.6 mg·kg^−1^·day^−1^ 1 : 4 mixture of lupenone and caryophyllene oxide. Statistical analysis was performed using one-way ANOVA and post hoc Tukey's test: *P < 0.05 compared with negative control.

**Figure 4:**
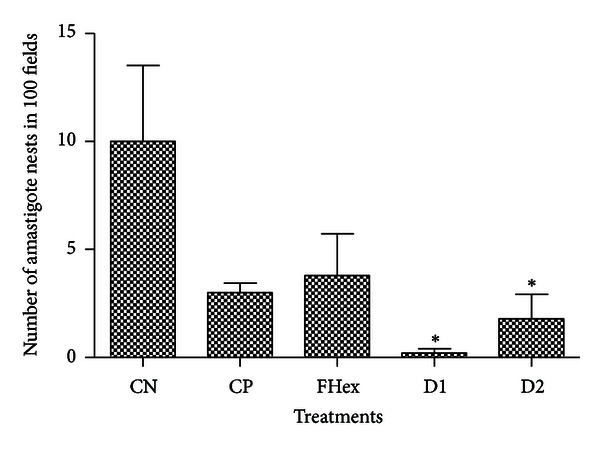
*In vivo* antitrypanosomal activity of the different treatments, determined by the number of amastigote nests observed in skeletal muscle of infected mice. CN: negative control, infected animals treated with PBS; CP: positive control, infected animals treated with allopurinol (8.5 *μ*g/g); FHex: hexane fraction, animals treated with the hexane fraction from the leaf crude extract of *S. yucatanensis* (41.6 mg·kg^−1^·day^−1^); D1: dose 1, infected mice treated with a 20.8 mg·kg^−1^·day^−1^ 1 : 4 mixture of lupenone and caryophyllene oxide; D2: dose 2, infected mice treated with a 41.6 mg·kg^−1^·day^−1^ 1 : 4 mixture of lupenone and caryophyllene oxide. Statistical analysis was performed using one-way ANOVA and post hoc Tukey's test: *P < 0.05 compared with negative control.

Finally, testing of the hexane fraction (41.6 mg·kg^−1^·day^−1^) and both doses of the 1 : 4 mixture of terpenoids for their capacity to reduce the number of amastigote nests in infected mice showed that both doses of the 1 : 4 mixture of lupenone and caryophyllene oxide reduced the presence of amastigote nests in cardiac tissue by more than 80% (*P* < 0.05) when compared to untreated mice (Figure 3). The 1 : 4 mixture of terpenoids also showed an important reduction in the number of amastigotes nests in skeletal muscle, with the lowest dose (20.8 mg·kg^−1^·day^−1^) showing a reduction of more than 98% (*P* < 0.05) when compared to the negative control (Figure 4). The activity of the 1 : 4 terpenoid mixture against amastigotes of *T. cruzi* is particularly important because most drugs presently used for the treatment of Chagas disease are effective mainly in the acute phase of the disease but not in the chronic phase (3–5).

It is interesting to point out that the hexane fraction, with *in vitro* activity against epimastigotes, showed no antitrypanosomal activity *in vivo* against amastigote of *T. cruzi* in both heart tissue and skeletal muscle. Additionally, although mice treated with the reference drug allopurinol showed a decrease in the number of amastigote nests in cardiac tissue and skeletal muscle, the value was not significantly different to that observed in untreated mice (Figures 3 and 4); furthermore, the mortality and physical deterioration observed in the animals treated with allopurinol might be due to the reported toxicity of the reference drug when used in the chronic phase of the disease [4, 5].

To date there are few reports on the use of synergism as a combined therapy against *T. cruzi*; these include the synergistic effect observed between amiodarone and posaconazole [28] and between aspirin and nifurtimox; the latter reported to be a consequence of the capacity of aspirin to increase the antiparasitic activity of macrophages [29]. A synergistic effect between parthenolide, a terpenoid isolated from *Tanacetum vulgare*, and benznidazole has also been confirmed [30], and the combination of benznidazole and ketoconazole is reported to act synergistically to inhibit the parasite in the acute phase of Chagas disease in mice infected with CL and Y strains of *T. cruzi* [31]. Taking into account that, to date, there is no adequate treatment for Chagas disease and that the number of studies on the use of synergism as a combined treatment strategy against *T. cruzi* and in the chronic phase of the disease is still limited, the synergistic effect shown by the 1 : 4 mixture of lupenone and caryophyllene oxide against *T. cruzi in vivo* represents an important option for the future use of two commercially available natural products to treat this parasitosis. 

## 4. Conclusions 

This is the first report on the trypanocidal activity of a mixture of lupenone and caryophyllene oxide against *T. cruzi in vitro* and *in vivo*. Our results showed that the 1 : 4 mixture of lupenone and caryophyllene oxide is active in the chronic phase of the disease, reducing significantly the number of amastigote nests in both cardiac tissue and skeletal muscle. Future studies will include the evaluation of the trypanocidal activity of the terpenoid mixture at lower concentrations and its administration to infected mice for longer periods of time, together with a better understanding of its mode of action.

## Figures and Tables

**Table 1:** *In vitro* trypanocidal activity (IC_50_ values in *μ*g/mL) of mixtures of lupenone and caryophyllene oxide.

Sample	*Trypanosoma cruzi* Tulahuen strain	FIC
SYH	74.5 *μ*g/mL	—
FHex	61.5 *μ*g/mL	—
Lupenone + caryophyllene oxide 1 : 0	85.0 *μ*g/mL	—
Lupenone + caryophyllene oxide 4 : 1	>100 *μ*g/mL	5.62
Lupenone + caryophyllene oxide 3 : 2	80.0 *μ*g/mL	3.59
Lupenone + caryophyllene oxide 1 : 1	86.5 *μ*g/mL	3.88
Lupenone + caryophyllene oxide **1** : **4**	**10.4** *μ*g/mL	**0.46**
Lupenone + caryophyllene oxide 2 : 3	39.8 *μ*g/mL	1.79
Lupenone + caryophyllene oxide 0 : 1	30.1 *μ*g/mL	—
Anfotericina B	0.7 *μ*g/mL	—

FIC: fractional inhibitory concentrations; SYH: leaf extract of *Serjania yucatanensis*; FHex: hexane fraction.
